# Radial simultaneous multi slice imaging for rapid cardiac imaging

**DOI:** 10.1186/1532-429X-18-S1-O111

**Published:** 2016-01-27

**Authors:** Ganesh Adluru, Erik Bieging, Liyong Chen, Daniel Kim, Brent D Wilson, Edward V DiBella

**Affiliations:** 1 Radiology, University of Utah, Salt lake city, UT USA; 2 Advaned MRI Techonologies, Berkeley, CA USA; 3 Cardiology, University of Utah, Salt Lake City, UT USA

## Background

It may be possible to more rapidly acquire cine, cardiac perfusion, and Late Gadolinium Enhancement (LGE) images for "free" with Simultaneous Multi-Slice (SMS) methods. With SMS imaging, multiple slices are simultaneously excited and acquired. Here we explore the application of an undersampled radial SMS approach termed Controlled Aliasing In Parallel Imaging results in higher acceleration (CAIPI) [1] combined with an iterative constrained reconstruction method [2] for rapid comprehensive cardiac imaging.

## Methods

Radial CAIPI data with golden ratio angular spacing [3] was acquired for cardiac perfusion and LGE imaging using a 32 channel cardiac coil on a Siemens 3T Verio scanner. ECG gated cardiac perfusion imaging at rest was performed using a saturation recovery sequence with TR = 2.6 msec, TE = 1.5 msec, matrix size = 128 × 30, field-of-view = 300 mm^2^, saturation recovery time = 70 msec, flip angle = 12°, slice acceleration/CAIPI factor = 3, slice thickness = 5 mm, spacing between slices = 6 mm. LGE images were acquired using an inversion recovery sequence and TR = 2.5 msec, TE = 1.4 msec, inversion recovery time = 350 msec, flip angle = 12°, slice acceleration factor=2, slice thickness = 8 mm, spacing between slices = 9.6 mm. Thirty rays were acquired every other heart beat in the diastolic cardiac phase until a total of 90 rays were acquired. Radial cardiac cine data without CAIPI was acquired with a temporal resolution of 43.55 msec, TE = 1.7 msec, flip angle = 50, field-of-view = 220 mm^2^, slice thickness = 5 mm, spacing between slices = 42 mm, matrix size = 128 × 30. CAIPI data with a slice acceleration factor = 2 was simulated using the acquired radial cine data.

Undersampled radial CAIPI data was reconstructed using an iterative spatio-temporal constrained reconstruction (STCR) framework in which data fidelity to the acquired CAIPI data was preserved while applying temporal Total Variation (TV) and spatial TV constraints on individual slices separately [2]. After coil compression [4], the coil sensitivities for the constrained reconstruction were computed using the CAIPI data by combining 250 rays after corresponding phase demodulation for each slice.

## Results

**Figure 1:**
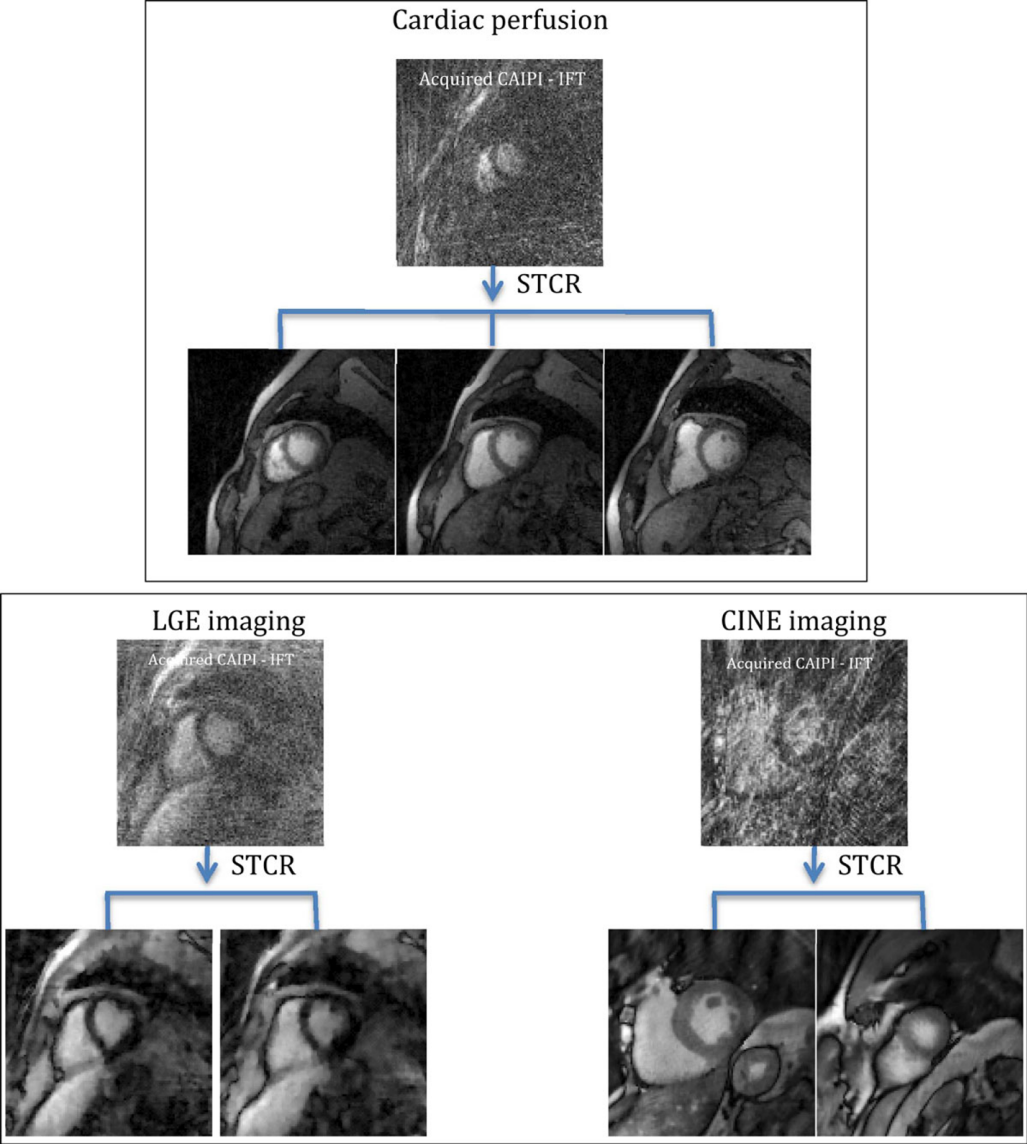
**Results of the radial CAIPI approach for cardiac perfusion, LGE and cine imaging**. Top row for each acquisition shows image reconstructed using inverse Fourier transform of the acquired CAIPI data. Joint multi-slice and multi-coil STCR reconstructions are shown below. One time framce from the dynamic sequence is shown for perfusion and cine acquisitions.

Figure 1 shows the results obtained using the radial CAIPI approach for cardiac perfusion, cine and LGE imaging. The image in the top row shows inverse Fourier Transform (IFT) reconstruction of the acquired CAIPI data obtained using NUFFT [5]. Images in the bottom row are obtained from the joint multi-slice and joint multi-coil iterative constrained reconstruction framework and are simultaneously excited.

## Conclusions

Simultaneously exciting multiple slices and using radial in-plane undersampling with constrained reconstruction techniques allows for significant speedups of 2D cine, perfusion, and LGE imaging.
